# Teaching Experience Correlates with Enhanced Social Cognition in Preschool Teachers

**DOI:** 10.3390/jintelligence14010010

**Published:** 2026-01-06

**Authors:** Daniela Molina-Mateo, Ivo Leiva-Cisterna, Paulo Barraza

**Affiliations:** 1 CIAE, Center for Advanced Research in Education, University of Chile, Santiago 8330014, Chile; daniela.molina@ciae.uchile.cl (D.M.-M.); ivo.leiva@ciae.uchile.cl (I.L.-C.); 2 IE, Institute for Advanced Studies in Education, University of Chile, Santiago 8330014, Chile

**Keywords:** teaching behavior, early childhood education, cognition, socio-affective skills, abstract reasoning

## Abstract

Preschool teaching is a highly demanding profession that requires constant socio-emotional attunement and the ability to engage in reflective reasoning. Despite the central role of these skills in effective early childhood education, little is known about whether preschool teachers’ socio-affective and cognitive capacities vary as a function of accumulated professional experience. To address this knowledge gap, we compared the performance of 30 professional preschool teachers with a matched control group of 30 non-teachers on tests measuring emotion recognition, active-empathic listening, interpersonal reactivity, and abstract reasoning. We found that preschool teachers were significantly better on all dimensions of active-empathic listening (sensing, processing, and responding) and better in emotional self-regulation than controls. Moreover, years of preschool teaching experience were positively correlated with emotion recognition, improved listening skills, and more deliberate abstract reasoning strategies. Notably, socio-affective competencies were correlated with abstract reasoning performance within the preschool teacher group. According to these results, long-term professional involvement in preschool teaching enhances socio-affective skills and integrates them with higher-order cognitive processes, both of which are essential for responsive teaching, efficient classroom management, and the development of children’s social and cognitive abilities.

## 1. Introduction

Teaching young children is one of the most socially and cognitively demanding human activities. Early childhood educators must decipher nuanced emotional cues, negotiate complicated classroom dynamics, and react in ways that promote participation and learning. It has been shown that children’s social and academic outcomes can be accurately predicted by the quality of teacher-student interactions (Mashburn et al., 2008; Justice et al., 2008; Commodari, 2013; Gerholm et al., 2019). Therefore, effective preschool teaching calls for more than just subject-matter expertise. Long-term socio-affective competence and the capacity to self-regulate emotions while maintaining empathy are required (Jennings & Greenberg, 2009; Pianta et al., 2012; Vorkapić & Ružić, 2013; Huang et al., 2020). Despite the evident importance of these skills, there are still few empirical studies comparing preschool teachers to non-teaching adults in the socio-affective and cognitive domains, which raises unanswered questions about how professional experience affects these abilities.

One interesting research line concerns the development of emotional self-regulation and active-empathic listening skills in preschool teachers. Previous studies suggest that teachers who can modulate their emotional responses create classrooms that are experienced as calm, supportive, and conducive to learning (Sutton et al., 2009; Clipa & Boghean, 2015; Lee et al., 2016; Koçyiğit & Sezer, 2024). On the other hand, the active-empathic listening skill allows preschool teachers to understand children’s needs and build trust, fostering higher rapport and social connection while reducing defensive responses among interlocutors (McNaughton et al., 2008; Weger et al., 2010; Gearhart & Bodie, 2011; Itzchakov et al., 2017; Hernández-Calderón & Lesmes-Silva, 2018; Kourmousi et al., 2018; Sobar et al., 2023). Recent studies indicate that sustained teaching experience enhances socio-affective skills, refining emotional regulation and interpersonal responsiveness through repeated engagement with classroom challenges (Xiao & Tian, 2023; Wang et al., 2023; Gebre et al., 2025). This suggests that teachers, particularly at the preschool level, may enhance their socio-affective skills through repeated practice over the years.

It is important to note that socio-affective processing is not an isolated skill but is deeply integrated with reasoning, reflecting the dynamic interplay between emotion and cognition (Pessoa, 2008; Rodriguez, 2013). In this regard, it has been proposed that emotional experiences shape reasoning and decision-making processes (Damasio, 1994; Blanchette & Richards, 2010), while socio-emotional engagement recruits neural circuits supporting abstract thinking and executive control (Immordino-Yang & Damasio, 2007; Bernhardt & Singer, 2012). In the case of teachers, it has been stated that they can adjust to changing classroom situations by integrating reflective problem-solving with affective sensitivity. For instance, Rodriguez and Fitzpatrick (2011) propose that, compared to novice teachers, expert teachers are characterized by being aware of cognitive and affective processes simultaneously during teaching. On the other hand, Barraza and Rodríguez (2023) report that expert teachers’ cognition stands out because their executive functions are more efficient, while they are more reflective during affective mentalization processes. Building on this, recent integrative frameworks argue that adaptive expertise relies on tightly coupled emotion–cognition dynamics (Brockbank & Feldon, 2024; Geng et al., 2024; Nejati, 2025), suggesting that socio-affective and reasoning abilities not only strengthen with professional experience but also become increasingly interdependent. Despite this compelling theoretical landscape, empirical comparisons between preschool teachers and non-teaching professionals remain scarce.

This study aims to examine whether professional preschool teachers show better performance in terms of their socio-affective (emotion recognition, active-empathic listening, and interpersonal reactivity) and abstract reasoning skills compared to a control group of adults without professional training in teaching. Importantly, to rule out educational or credentialing confounds, the present study compares preschool teachers with a control group of university-educated professionals from non-teaching fields, ensuring comparable academic attainment between groups. Also, both groups were matched on age and years of professional experience, allowing us to attribute group differences to the specific demands of preschool teaching rather than to general professional seniority or age-related factors. Considering the evidence mentioned earlier, we put forth the following hypotheses: (a) Preschool teachers are expected to exhibit higher levels of emotion recognition, active-empathic listening, interpersonal reactivity, and abstract reasoning skills compared to a control group; (b) Socio-affective and abstract reasoning skills are expected to increase as a function of years of preschool teaching experience; (c) Socio-affective and abstract reasoning processes are expected to be more strongly correlated in preschool teachers than in a control group. To test these hypotheses, we compared the behavioral responses of professional preschool teachers with those of a demographically matched cohort of non-teaching professional adults across four cognitive and socio-affective assessments (Davis, 1983; Baron-Cohen et al., 2001; Drollinger et al., 2006; Bodie, 2011; Raven et al., 2012). According to our hypotheses, we expected that professional preschool teachers would exhibit superior socio-affective and abstract reasoning skills compared to the control group, and that these abilities would improve with years of preschool teaching experience, suggesting a potential enhancement of social-cognitive processes through sustained professional practice of preschool teaching.

## 2. Materials and Methods

### 2.1. Participants

This study evaluated 60 women, 30 preschool teachers (age range = 28–61 years, mean = 39.63 years, SE = 1.38), and 30 non-preschool teachers (age range = 31–64 years, mean = 39.70 years, SE = 1.34). The preschool teachers had an average of 13.63 years of professional experience (range = 5–33 years, SE = 1.24), while the control group had an average of 14.30 years of professional experience in their respective jobs (range = 6–42 years, SE = 1.36). The control group was recruited by matching the preschool teacher group in terms of age (t_(58)_ = −0.035, *p* = .972, Cohen’s d = −0.008), years of professional experience (t_(58)_ = −0.362, *p* = .719, Cohen’s d = −0.093), socioeconomic status (t_(58)_ = −1.433, *p* = .157, Cohen’s d = −0.370), and number of children (t_(58)_ = 0.691, *p* = .492, Cohen’s d = 0.179). The fact that the sample is composed exclusively of women reflects the demographic reality of early childhood education in our national context, where women constitute more than 99% of the workforce (MINEDUC, 2025). Thus, the gender composition of our sample mirrors the population from which preschool teachers can be recruited. Importantly, gender was not used as a sampling criterion.

“Preschool teachers” (PST) were defined as adults holding a university degree in early childhood education and employed full-time in preschool settings. “Non-preschool teachers” (CNT) were defined as adults with university degrees in any field other than pedagogy or early childhood education, who had never taught in educational contexts. The CNT group encompassed a deliberately heterogeneous set of professions, including nursing (5), engineering (5), biochemistry (3), psychology (2), architecture (2), medicine (1), geography (1), design (1), accounting and auditing (1), social work (1), anthropology (1), dentistry (1), midwifery (1), law (1), history (1), archeology (1), journalism (1), and biotechnology (1). Notably, the professional field was not an exclusion criterion. Instead, we deliberately recruited CNT participants from a broad range of occupations to mitigate profession-specific effects and avoid systematic biases associated with any single career path. Crucially, our design does not assume that CNT professions lack interpersonal or sociocognitive demands; instead, it evaluates whether the distinctive relational ecology of preschool teaching is associated with a differentiated socio-affective profile relative to a heterogeneous non-teaching cohort. All participants reported normal or corrected vision and hearing, no history of neuropsychiatric or neurological conditions, and Spanish as their native language. Each participant read and signed an informed consent form before beginning the study. The study was approved by the Ethics Committee for Research in Social Sciences and Humanities of the Faculty of Philosophy and Humanities at the University of Chile.

### 2.2. Research Instruments

#### 2.2.1. Reading the Mind in the Eyes Test (RMET-R)

We used the RMET-R (Baron-Cohen et al., 2001) to evaluate the subjects’ ability to recognize emotions and mental states in others. The task includes 36 black-and-white photos of people’s eye regions, featuring 19 males and 17 females. Each image is paired with four words that describe different emotional states, such as “Ashamed,” “Nervous,” “Suspicious,” and “Indecisive”. The participants’ task was to select the word that most accurately characterized the individual depicted in each picture. Prior to the experiment, participants read the instructions to perform the task and then performed one practice trial. Each trial began with a blank screen (0.2 s), followed by one of the images and four single-word descriptors, which remained on the screen until the participant answered. The participant had to click on one of the four words with the mouse to respond. For further analysis, we calculated the average accuracy percentage and the average response time (RT) for correct responses during the entire task to measure overall performance.

#### 2.2.2. Interpersonal Reactivity Index (IRI)

To assess the participants’ dispositional tendencies toward empathy, we used the Interpersonal Reactivity Index (Davis, 1983), which assessed four distinct, established dimensions of empathy: Perspective Taking, Empathic Concern, Fantasy, and Personal Distress. This tool consists of 28 items, with seven items in each category. Each item is rated on a 5-point Likert-type scale that goes from 1, which means “Does not describe me well,” to 5, which means “Describes me very well”. All items were shown on printed questionnaires. Participants marked their responses directly on the response sheet with a pencil. The task was self-paced and had no time limits. Before beginning, participants received verbal instructions and could ask questions for clarification if necessary. For further analysis, we calculated subscale scores by adding the response ratings within each category. We also computed a total score by adding the scores of all 28 items.

#### 2.2.3. Active-Empathic Listening Scale (AELS)

We used the Active and Empathic Listening Scale (Drollinger et al., 2006; Bodie, 2011) to assess participants’ self-perceived listening abilities across three dimensions: Sensing, Processing, and Responding. The AELS consists of 11 items rated on a 7-point Likert-type scale ranging from 1 (“Never or almost never true”) to 7 (“Always or almost always true”). Items were presented in a printed questionnaire, and participants indicated their responses by circling the appropriate number on the response sheet. The task was self-paced, and no time limit was imposed. Before the task, participants received verbal instructions and were allowed to ask clarifying questions if needed. For analysis, we computed mean scores for each of the three subscales by averaging the ratings to their respective items. In addition, we calculated a total AELS score by averaging responses across all 11 items, providing an overall index of active-empathic listening ability.

#### 2.2.4. Raven Progressive Matrices (RPM)

We used the Raven Progressive Matrices (Raven, 1938; Raven et al., 2012) to assess participants’ non-verbal abstract reasoning and fluid intelligence. The task consists of 36 items, each presenting a matrix of geometric patterns with one missing element. For each item, participants must select the correct piece, out of eight options, that completes the pattern according to the underlying logical rule. Each trial consists of presenting the incomplete pattern and the eight response options below. The stimulus remained on the screen until the participant responded by clicking on one of the options using the mouse. Participants were instructed to work as accurately and quickly as possible. Before beginning the test, they read on-screen instructions and completed one untimed practice trial to familiarize themselves with the task. For analysis, we computed the total accuracy score (number of correct responses) and the mean response time (RT) for correct responses as indicators of abstract reasoning performance.

### 2.3. Procedures

Participants sat comfortably at a desk about 63 ± 3 cm from the screen of a personal computer (HP Pavilion Notebook, 14). No other electronic devices were on the desk, and mobile phones stayed turned off during the experimental session. Participants received earplugs to reduce possible distractions from background noise. Before beginning, they read instructions for each task, and the experimenter addressed any questions. The experimental session included computer-based tasks (RPM and RMET) and paper-and-pencil questionnaires (IRI and AELS). The order of task modality was counterbalanced across participants. Participants interacted only with the screen and mouse when they performed the computerized tasks. The computer was closed and not in use when completing the paper-based tasks. Computer-based tasks were programmed and presented using E-Prime 3.0.3.80 (Psychology Software Tools, Inc., Sharpsburg, PA, USA). Paper-based tasks were completed using printed questionnaires, and participants recorded their answers with a pencil. Each task lasted approximately 7–10 min.

### 2.4. Statistical Analysis

Before comparing the means between the preschool teacher and control groups, the normality assumption was assessed using the Shapiro–Wilk test. A one-way ANOVA was used to perform the group comparisons. The significance level was set at 0.05. Effect size was calculated using omega squared (ω^2^). Additionally, Pearson correlations were used to assess the relationship between variables. All statistical analyses were performed using GraphPad Prism software version 11 (www.graphpad.com, accessed on 25 November 2025) and the free JASP software version 0.19 (https://jasp-stats.org/, accessed on 25 November 2025).

## 3. Results

### 3.1. Emotion Recognition

**Figure 1:**
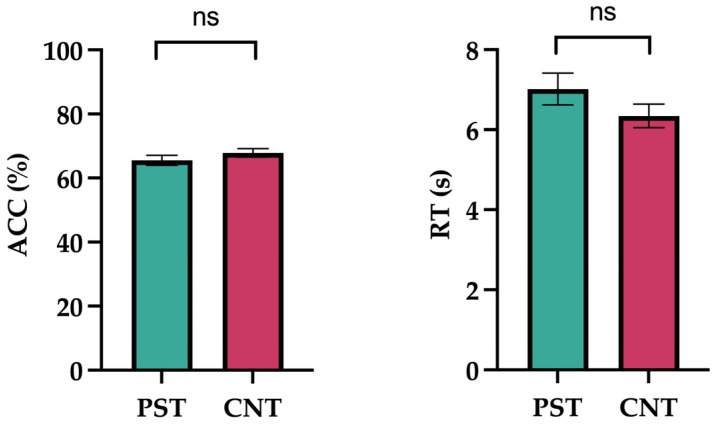
Reading the Mind in the Eyes task (RMET). Average accuracy percentage (ACC) and response time (RT) in seconds are presented on the *y*-axis, while the groups are indicated on the *x*-axis. Error bar indicates SEM. ns = non-significant.

The results are illustrated in Figure 1. Performance on the RMET revealed that the mean ACC in the preschool teacher was 65.56% (SE = 1.56) with a mean RT of 7.02 s (SE = 0.40), whereas in the control group it was 67.87% (SE = 1.30) with a mean RT of 6.34 s (SE = 0.29). A one-way ANOVA indicated no significant between-group differences in either accuracy (*F*_(1,58)_ = 1.306, *p* = .258, ω^2^ = 0.005) or response time (*F*_(1,58)_ = 1.839, *p* = .180, ω^2^ = 0.014).

### 3.2. Interpersonal Reactivity

**Figure 2:**
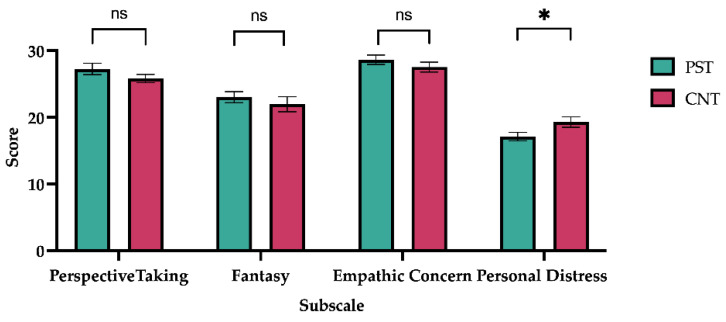
Interpersonal Reactivity Index (IRI). Average score is presented on the *y*-axis, while the subscales are indicated on the *x*-axis. Green and pink bars represent preschool teachers (PST) and control group (CNT), respectively. Error bar indicates SEM. ns = non-significant; * *p* < .05.

The results are illustrated in Figure 2. Performance on the IRI revealed that the mean scores by Perspective Taking, Fantasy, Empathic Concern, and Personal Distress subscales for the preschool teacher group were 27.27 (SE = 0.86), 28.63 (SE = 0.70), 23.03 (SE = 0.83), and 17.13 (SE = 0.64) points, respectively, while the mean scores for the control group were 25.83 (SE = 0.61), 21.97 (SE = 1.12), 27.53 (SE = 0.74), and 19.30 (SE = 0.78) points, respectively. A one-way ANOVA revealed a significant between-group difference in the Personal Distress subscale (*F*_(1,58)_ = 4.653, *p* = .035, ω^2^ = 0.057). No significant differences emerged for the remaining subscales: Perspective Taking (*F*_(1,58)_ = 1.839, *p* = .180, ω^2^ = 0.014), Fantasy (*F*_(1,58)_ = 0.587, *p* = .447, ω^2^ = 0) and Empathic Concern (*F*_(1,58)_ = 1.159, *p* = .286, ω^2^ = 0.003).

### 3.3. Active-Empathic Listening

**Figure 3:**
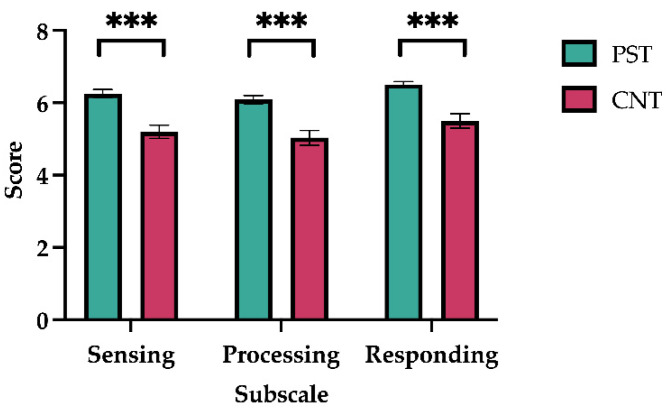
Active-Empathic Listening Scale (AELS). Average score is presented on the *y*-axis, while the subscales are indicated on the *x*-axis. Green and pink bars represent preschool teachers (PST) and control group (CNT), respectively. Error bar indicates SEM. *** *p* < .0001.

The results are illustrated in Figure 3. Performance on the AELS revealed that the mean scores by Sensing, Processing and Responding subscales for the preschool teacher group were 6.40 (SE = 0.13), 6.10 (SE = 0.12), and 6.63 (SE = 0.10) points, respectively, while the mean scores for the control group were 5.3 (SE = 0.20), 5.10 (SE = 0.19), and 5.67 (SE = 0.20) points, respectively. A one-way ANOVA revealed a significant between-group difference in all subscale: Sensing (*F*_(1,58)_ = 25.157, *p* = 5.309 × 10^−6^, ω^2^ = 0.287), Processing (*F*_(1,58)_ = 21.044, *p* = 2.448 × 10^−5^, ω^2^ = 0.250), and Responding (*F*_(1,58)_ = 22.081, *p* = 1.652 × 10^−5^, ω^2^ = 0.260).

### 3.4. Fluid Intelligence

**Figure 4:**
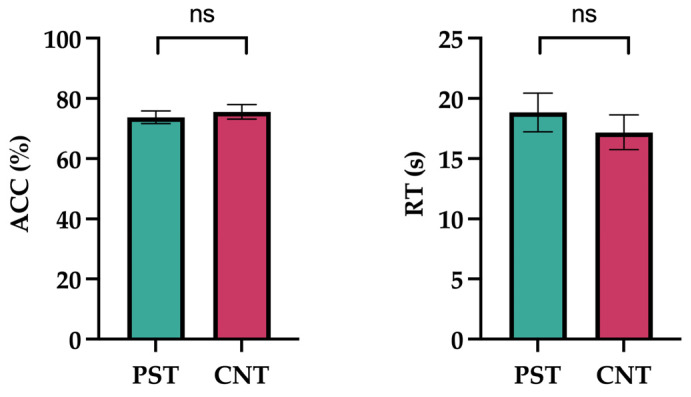
Raven Progressive Matrices (RPM). Average accuracy percentage and response time in seconds are presented on the *y*-axis, while the groups are indicated on the *x*-axis. Error bar indicates SEM. ns = non-significant.

The results are illustrated in Figure 4. Performance on the RPM revealed that the mean ACC in the preschool teacher group was 73.70% (SE = 2.13) with a mean RT of 19.00 s (SE = 1.60), whereas in the control group it was 75.60% (SE = 2.40) with a mean RT of 17.17 s (SE = 1.45). A one-way ANOVA indicated no significant between-group differences in either accuracy (*F*_(1,58)_ = 0.317, *p* = .576, ω^2^ = 0) or response time (*F*_(1,58)_ = 0.590, *p* = .446, ω^2^ = 0).

### 3.5. Correlation Analysis

The results are illustrated in Table 1. As for preschool teacher, a bivariate Pearson correlation analysis revealed an association between the years of teaching experience and RMET accuracy (r = 0.434, *p* = .017), AELS processing score (r = 0.418, *p* = .022), and RPM response time (r = 0.556, *p* = .001). No other correlations with years of teaching experience reached significance. For the control group, no correlations with years of professional experience reached significance.

Additionally, we examined correlations between socio-affective and cognitive measures in preschool teachers and control (Table 2). In preschool teachers, RPM response time was positively associated with AELS scores (sensing: r = 0.409, *p* = .025; processing: r = 0.403, *p* = .027; responding: r = 0.398, *p* = .029). Higher IRI Personal Distress was linked to lower AELS scores (sensing: r = –0.593, *p* = .0005; processing: r = –0.394, *p* = .031; responding: r = –0.396, *p* = .030) and slower RPM responses (r = –0.505, *p* = .004). RMET response time also correlated positively with RPM response time (r = 0.376, *p* = .040) and accuracy (r = 0.445, *p* = .014). No other correlations reached significance. In the control group, IRI Perspective Taking correlated with AELS sensing (r = 0.406, *p* = .026) and RPM accuracy (r = 0.433, *p* = .017), whereas IRI Empathic Concern was associated with AELS sensing (r = 0.415, *p* = .023). All other correlations were non-significant.

## 4. Discussion

This study contrasted the performance of professional preschool teachers with that of a control group of non-preschool teachers on tasks and questionnaires assessing affective mentalization, interpersonal reactivity, active–empathic listening, and fluid intelligence. The results revealed that preschool teachers outperformed controls in emotional self-regulation and active-empathic listening. Moreover, years of preschool teaching experience were linked to better emotion recognition, active-empathic listening, and deliberative abstract reasoning. A functional connection appeared among emotional self-regulation, active-empathic listening, and abstract reasoning in the preschool teacher group. Below, we elaborate on the main findings and their broader implications.

### 4.1. Preschool Teachers Show Enhanced Emotional Self-Regulation and Active-Empathic Listening

Preschool teachers showed a different emotional self-regulation and listening skills profile when contrasted with the control group. Lower scores on the IRI Personal Distress subscale indicated that they were less likely to feel uncomfortable when faced with the distress of others. At the same time, higher scores on the AELS sensing, processing, and responding dimensions reflected more efficient perception, integration, and behavioral expression of empathic listening. Together, these findings suggest that preschool teachers engage in affective empathy in a way that privileges understanding and responsiveness to others over self-oriented affective reactions. Such a profile is consistent with prior work linking lower personal distress to more adaptive empathic concern (Eisenberg & Eggum, 2009; Hall & Schwartz, 2019; Schwarzer et al., 2023) and associating active-empathic listening with greater trust, rapport, and prosocial engagement (Weger et al., 2014; Itzchakov et al., 2018; Hernández-Calderón & Lesmes-Silva, 2018; Kourmousi et al., 2018). Preschool teachers need these abilities to control classroom dynamics, pick up on young children’s subtle emotional cues, and establish encouraging learning environments. For example, preschool teachers frequently have better emotional self-control when faced with an emotionally charged situation, such as a child becoming frustrated while working on a task. This helps them stay focused on the child’s perspective and respond helpfully. These skills help maintain a calm and focused classroom climate and promote the development of children’s emotional and communicative competencies through modeling and responsive engagement.

Building on this behavioral profile, it is reasonable to consider that these advantages in emotional regulation and active-empathic listening may not only manifest in observable interactional competencies but may also be supported by underlying neurocognitive mechanisms that work in coordination. In other words, the differences we identify at the behavioral level are likely grounded in neurocognitive systems that perform distinct yet complementary functions. Emotional self-regulation is typically associated with networks linking prefrontal regions to structures involved in affective responding, which together enable individuals to maintain composure and flexibly adjust their reactions in challenging situations (Shamay-Tsoory et al., 2003; Etkin et al., 2015). In parallel, active-empathic listening engages circuits implicated in attending to social cues, interpreting communicative intent, and adopting others’ perspectives—capacities central to understanding and responding to others in meaningful ways (Stephens et al., 2010; Zaki & Ochsner, 2012; Shany et al., 2021). In everyday classroom practice, these systems are likely co-activated: regulating one’s emotional responses while simultaneously attending to the child’s signals supports a more attuned and sustained instructional interaction. Understanding how ongoing teaching experience shapes the coordination of these neurocognitive systems represents a promising direction for future research.

### 4.2. Relationship of Teaching Experience with Emotion Recognition, Active Listening, and Abstract Reasoning

Our results show that, among preschool teachers, years of teaching experience were positively associated with better emotion recognition accuracy, active-empathic listening, and slower response speed in abstract reasoning. No significant correlations emerged in the control group. This result pattern is consistent with evidence showing that long-term immersion in emotionally demanding teaching environments enhances interpersonal sensitivity and socio-emotional accuracy (Carmen et al., 2022; Xiao & Tian, 2023), suggesting that repeated engagement with children’s affective cues naturally strengthens emotion-perception mechanisms. Likewise, the association between increased experience and more reflective cognitive responses aligns with models indicating that expert practitioners increasingly rely on controlled, evaluative processing to navigate complex classroom situations (Wolff et al., 2021; Miller et al., 2024). Further, the fact that the response times in abstract reasoning tasks became slower, are in line with a more thoughtful approach to solving complex problems, as proposed by Cunningham and Zelazo (2007) and Cunningham et al. (2007), who argue that slower responses reflect a more conscious and reflective evaluative process. As for the interpretation of these results in the early childhood education context, we propose that the emergent demands of preschool teaching, such as understanding children’s emotions, paying close attention to verbal and non-verbal signals, and effectively addressing instructional challenges, gradually improve emotional sensitivity and reasoning strategies (Huang et al., 2020; Vučinić et al., 2020). Evidence that intensive, specialized professional experience improves socio-affective skills and deliberate thinking supports this idea (Eisenberg & Eggum, 2009; Gross, 2015; Guerra-Zamora et al., 2017; Valente et al., 2022). It is essential to note that, given the absence of these associations in the control group, the pattern observed in preschool teachers is unlikely to be a mere age-related change. Instead, the graded relationship between years of teaching and performance suggests a domain-specific training effect shaped by sustained professional experience.

An alternative hypothesis is a selection effect, namely, individuals who already have a specific socio-affective and cognitive profile, marked by strong emotion recognition, good listening skills, and a thoughtful way of solving problems, are more likely to enter and stay in the teaching profession. Studies have shown that teachers with stronger interpersonal competencies are less likely to leave teaching prematurely (Masari et al., 2013; Vučinić et al., 2020; Goldenberg, 2024) and that prospective educators who are more empathic and with prosocial motivation are more likely to choose teaching as a career (Li & Guo, 2024). Distinguishing between training and selection effects hypothesis requires longitudinal studies assessing teachers before entering the profession and tracking developmental trajectories across pre-service training and early career stages. Such designs could clarify whether enhanced affective and cognitive profiles are primarily a cause or consequence of teaching experience, or a combination of both.

### 4.3. Functional Interplay Between Emotional Self-Regulation, Active Listening, Complex Emotion Recognition and Abstract Reasoning in Preschool Teachers

Our data reveal that, among preschool teachers: (i) slower response speed in abstract reasoning was associated with better active–empathic listening, emotional self-regulation, and complex emotion recognition, (ii) better emotional self-regulation was associated with better active–empathic listening, and (iii) slower response speed in complex emotion recognition was associated with better abstract reasoning. These findings align with previous research, which has shown a correlation between more reflective modes of cognitive processing and more accurate socio-emotional judgments, deeper empathic engagement, and improved emotion regulation (Smith et al., 2022; Double & Cavanagh, 2024; Yapıcı & Uzer, 2025). Notably, the reciprocal links among emotional self-regulation, empathic listening, and deliberate abstract reasoning suggest that socio-affective attunement does more than merely dampen emotional reactivity; instead, it provides a scaffold for complex cognition by integrating affective resonance with reflective interpretation (Decety & Jackson, 2004; Decety & Jackson, 2006; Immordino-Yang & Damasio, 2007; Frith & Frith, 2012; Bernhardt & Singer, 2012). Complementarily, the association between more deliberate complex emotion recognition with higher accuracy and more reflective responses in abstract reasoning tasks, suggesting that socio-affective engagement may scaffold higher-order reasoning by recruiting integrative cognitive mechanisms that bridge emotion and reasoning (Immordino-Yang & Damasio, 2007; Pessoa, 2008; Rodriguez, 2013; Shany et al., 2021). This pattern aligns with evidence that socially and cognitively demanding roles enhance domain-general executive resources and abstract thought (Singer & Lamm, 2009), highlighting a bidirectional interplay between empathic attunement and efficient decision-making in complex environments (Singer & Lamm, 2009; Frith & Frith, 2012; Tomasello, 2019).

As for the role played by this integrated system of socio-affective processes and abstract thinking during preschool teaching, we propose that these processes support complex pedagogical demands while maintaining empathic responsiveness (Garner, 2010; Ye et al., 2024). In particular, they appear to coactivate and integrate the higher-order cognitive operations that bridge affective and reasoning processing (Singer & Lamm, 2009; Tomasello, 2019; Hall & Schwartz, 2019; Barraza & Rodríguez, 2023), underscoring the possibility that relational engagement may function as a cognitive enhancer within educational contexts (Pessoa, 2008). For instance, when preschool teachers interact with children, they put into action emotion self-regulation to maintain a calm and supportive classroom climate, even in the face of disruptive or emotionally charged situations, active listening to capture verbal and non-verbal cues, emotion recognition to decode feelings and intentions accurately, and abstract reasoning to solve instructional problems, integrate past observations with new information, and anticipate potential learning challenges. A professional refinement of socio-cognitive integration developed through long-term pedagogical practice is reflected in these experiences, which continuously engage social, emotional, and cognitive resources and call for a finely tuned network coordinating empathic attunement, perceptual sensitivity, and reflective problem-solving. The longitudinal development of these networks, their neural substrates, and whether these integrated processes predict the learning outcomes of children and the efficacy of preschool instruction should all be the focus of future research.

### 4.4. Limitations

A further limitation concerns the gender composition of the sample. Because all participants were women, a pattern that reflects the extreme gender segregation of the early childhood education workforce in our national context (MINEDUC, 2025), the generalizability of the findings to male preschool teachers remains uncertain. Although gender was not a sampling criterion, and the sample accurately mirrors the population from which preschool teachers can be recruited, future studies should include more gender-diverse cohorts to determine whether the socio-affective and reasoning patterns observed here extend beyond this predominantly female professional group. Additionally, our comparison group did not allow us to distinguish preschool teaching from other high-interaction professions (e.g., nursing, social work, clinical psychology). Addressing this question will require dedicated cross-professional studies capable of mapping how different interpersonal ecologies shape socio-affective and cognitive skills. Finally, recruiting a diverse control group that matches preschool teachers on key demographic variables and encompasses a range of professions enhances the design. This approach avoids biases associated with specific professions and enhances the real-world relevance of the comparison. However, since we only matched the control group based on demographics and not on motivational factors that could influence individuals’ choices regarding early childhood education, some unaccounted confounding variables may still exist. Future studies with larger sampling pools could implement propensity score matching to construct more rigorously comparable control groups and further mitigate unmeasured confounding.

## 5. Conclusions

This study reveals that preschool teachers exhibit a unique combination of socio-emotional skills that appears to strengthen as a function of accumulated professional experience. These capacities do not operate in isolation. Instead, they form a coordinated network in which emotional self-regulation, active-empathic listening, emotion recognition, and deliberate problem-solving mutually reinforce each other. Integrating these processes would help preschool teachers resolve complex classroom problems, interpret subtle social and emotional signals, and respond adaptively to the challenges of guiding young children. Interestingly, the deliberate and sustained teaching practice in socio-affectively intense classroom environments seems to mold the coactivation of the emotional and cognitive systems. The above points to a cross-fertilization between expertise in early childhood education and fostering the dynamic integration of both capacities. Finally, these findings suggest practical avenues for strengthening early childhood education: professional development programs that cultivate emotion regulation, empathic listening, and reflective reasoning may help consolidate the socio-cognitive profile associated with effective preschool teaching. Although causal inferences cannot be drawn from this correlational design, the present results indicate that these competencies may be responsive to structured pedagogical practice, warranting future intervention studies to assess their trainability.

## Figures and Tables

**Table 1:** Correlations Between Years of Experience and Cognitive and Socio-Affective Measures in Preschool Teachers and Controls.

Cognitive Performance	PST		CNT	
	Pearson’s r	*p*-Value	Pearson’s r	*p*-Value
RPM ACC	0.192	.310	−0.045	.812
RPM RT	**0.556**	**.001**	0.350	.058
RMET ACC	**0.434**	**.017**	0.246	.190
RMET RT	0.310	.096	0.168	.375
AELS sensing	0.123	.516	−0.004	.984
AELS processing	**0.418**	**.022**	−0.216	.252
AELS responding	0.138	.466	0.121	.525
IRI perspective taking	0.201	.286	−0.159	.402
IRI fantasy	0.019	.919	−0.235	.210
IRI empathic concern	0.281	.133	−0.167	.378
IRI personal distress	−0.117	.538	−0.156	.410

Note. PST = preschool teachers; CNT = control group; ACC = accuracy; RT = response time; RPM = Raven’s Progressive Matrices; RMET = Reading the Mind in the Eyes Test; AELS = Active-Empathic Listening Scale; IRI = Interpersonal Reactivity Index. Significant correlations are shown in bold.

**Table 2:** Correlations Among Cognitive and Socio-Affective Measures in Preschool Teachers and Controls.

Cognitive Task	PST		CNT	
	Pearson’s r	*p*-Value	Pearson’s r	*p*-Value
RPM ACC				
RMET ACC	0.262	.162	0.224	.234
RMET RT	**0.445**	**.014**	0.132	.488
AELS sensing	0.111	.558	0.079	.677
AELS processing	−0.089	.641	0.084	.658
AELS responding	−0.036	.848	−0.219	.244
IRI perspective taking	0.181	.338	**0.433**	**.017**
IRI fantasy	−0.072	.704	0.182	.336
IRI empathic concern	0.195	.301	0.132	.485
IRI personal distress	−0.050	.793	−0.196	.299
RPM RT				
RMET ACC	0.236	.209	0.345	.062
RMET RT	**0.376**	**.040**	0.152	.423
AELS sensing	**0.409**	**.025**	0.156	.412
AELS processing	**0.403**	**.027**	−0.076	.692
AELS responding	**0.398**	**.029**	0.064	.739
IRI perspective taking	0.314	.091	0.133	.484
IRI fantasy	−0.190	.314	0.208	.270
IRI empathic concern	0.311	.095	0.228	.226
IRI personal distress	**−0.505**	**.004**	0.155	.413
RMET ACC				
AELS sensing	0.044	.817	−0.011	.952
AELS processing	0.056	.770	−0.264	.159
AELS responding	0.002	.990	−0.112	.556
IRI perspective taking	0.086	.652	−0.197	.296
IRI fantasy	0.105	.579	0.093	.623
IRI empathic concern	0.008	.968	−0.016	.931
IRI personal distress	0.037	.845	0.232	.218
RMET RT				
AELS sensing	−0.017	.929	0.047	.804
AELS processing	0.032	.867	−0.194	.305
AELS responding	−0.141	.458	0.029	.877
IRI perspective taking	0.243	.196	−0.101	.594
IRI fantasy	−0.090	.637	−0.079	.680
IRI empathic concern	0.235	.212	−0.032	.865
IRI personal distress	−0.056	.770	−0.236	.209
AELS sensing				
IRI perspective taking	0.242	.197	**0.406**	**.026**
IRI fantasy	−0.0001	1.000	0.159	.402
IRI empathic concern	0.125	.512	**0.415**	**.023**
IRI personal distress	**−0.593**	**.0005**	0.084	.660
AELS processing				
IRI perspective taking	0.282	.131	0.155	.412
IRI fantasy	−0.124	.513	−0.153	.419
IRI empathic concern	−0.010	.958	−0.040	.833
IRI personal distress	**−0.394**	**.031**	−0.165	.384
AELS responding				
IRI perspective taking	0.287	.124	0.065	.734
IRI fantasy	0.062	.746	0.212	.261
IRI empathic concern	0.143	.452	0.269	.151
IRI personal distress	**−0.396**	**.030**	0.094	.622

Note. PST = preschool teachers; CNT = control group; ACC = accuracy; RT = response time; RPM = Raven’s Progressive Matrices; RMET = Reading the Mind in the Eyes Test; AELS = Active-Empathic Listening Scale; IRI = Interpersonal Reactivity Index. Significant correlations are shown in bold.

## Data Availability

The data presented in this study are available on request from the corresponding author due to privacy ethical reasons.

## Abbreviations

The following abbreviations are used in this manuscript:

RMET	Reading the Mind in the Eyes Test
IRI	Interpersonal Reactivity Index
AELS	Active-Empathic Listening Scale
RPM	Raven Progressive Matrices
